# Leaf to panicle ratio (LPR): a new physiological trait indicative of source and sink relation in japonica rice based on deep learning

**DOI:** 10.1186/s13007-020-00660-y

**Published:** 2020-08-26

**Authors:** Zongfeng Yang, Shang Gao, Feng Xiao, Ganghua Li, Yangfeng Ding, Qinghua Guo, Matthew J. Paul, Zhenghui Liu

**Affiliations:** 1 College of Agriculture, Nanjing Agricultural University, Nanjing, 210095 China; 2 State Key Laboratory of Vegetation and Environmental Change, Institute of Botany, Chinese Academy of Sciences, Beijing, 100093 China; 3 Plant Science, Rothamsted Research, Harpenden, Hertfordshire AL5 2JQ UK; 4 Collaborative Innovation Center for Modern Crop Production, Nanjing Agricultural University, Nanjing, 210095 China

**Keywords:** Plant phenotyping, Leaf and panicle detection, Deep learning, Physiological trait, Leaf to panicle ratio (LPR), Japonica rice

## Abstract

**Background:**

Identification and characterization of new traits with sound physiological foundation is essential for crop breeding and production management. Deep learning has been widely used in image data analysis to explore spatial and temporal information on crop growth and development, thus strengthening the power of identification of physiological traits. Taking the advantage of deep learning, this study aims to develop a novel trait of canopy structure that integrate source and sink in japonica rice.

**Results:**

We applied a deep learning approach to accurately segment leaf and panicle, and subsequently developed the procedure of GvCrop to calculate the leaf to panicle ratio (LPR) of rice canopy during grain filling stage. Images of training dataset were captured in the field experiments, with large variations in camera shooting angle, the elevation and the azimuth angles of the sun, rice genotype, and plant phenological stages. Accurately labeled by manually annotating the panicle and leaf regions, the resulting dataset were used to train FPN-Mask (Feature Pyramid Network Mask) models, consisting of a backbone network and a task-specific sub-network. The model with the highest accuracy was then selected to check the variations in LPR among 192 rice germplasms and among agronomical practices. Despite the challenging field conditions, FPN-Mask models achieved a high detection accuracy, with Pixel Accuracy being 0.99 for panicles and 0.98 for leaves. The calculated LPR displayed large spatial and temporal variations as well as genotypic differences. In addition, it was responsive to agronomical practices such as nitrogen fertilization and spraying of plant growth regulators.

**Conclusion:**

Deep learning technique can achieve high accuracy in simultaneous detection of panicle and leaf data from complex rice field images. The proposed FPN-Mask model is applicable to detect and quantify crop performance under field conditions. The newly identified trait of LPR should provide a high throughput protocol for breeders to select superior rice cultivars as well as for agronomists to precisely manage field crops that have a good balance of source and sink.

## Background

Sustainable improvement in crop production is crucial for supporting the demand from an increasing global population, particularly considering that there are 821 M people who lack sufficient food to support their daily lives [1]. Recent technological advances in genome biology like next-generation sequencing, genome editing and genomic selection have paved the way for crop breeders to identify, characterize, transfer, or modify the genes responsible for grain yield or quality traits in a rapid and precise way [2]. However, there is a huge gap between the fundamental plant sciences and the applied science of crop breeding, as reflected by the limited understanding of the link between genotype and phenotype. Crop physiology is a key interface between the genome and the plant phenotype, and thus is indispensable for hastening crop improvement [3]. Accordingly, physiological breeding, a methodology for selection of physiological traits such as canopy temperature, carbon isotope discrimination, and stomatal conductance, was proposed. This approach has advantages over conventional breeding such as in water stressed Australian environments and in heat and drought stressed conditions of the International Wheat Improvement Network [4].

Chemically, cereal grain yield consists of photosynthetic assimilates first produced in the leaf source organs which are translocated to the sink organ of grain. Therefore, source and sink relations, the core concept of crop physiology, is the critical factor dominating crop yield formation. Improving the source activity of leaf photosynthesis to harness light irradiation more efficiently is one of the major targets of crop breeding. In rice, ideotypes have long been pursued by breeders, resulting in several successfully implemented theories such as the New Plant Type, Super Rice, and Ideal Plant Architecture [5–7]. One common future shared by these new plant types is the emphasis on leaf erectness, especially the top three leaves, which is supposed to be essential for improving source activity. However, some of the main cultivars with this ideotype had problems of incomplete filling of inferior grains, especially for those with large numbers of grains, indicating the importance of optimization of the source-sink ratio [8, 9].

In addition to storing photosynthetic assimilates from leaves, sink organs like glumes and awns have photosynthetic activity. Cumulative evidence favors the sizable contribution of spike or panicle to grain filling in terms of providing carbohydrates as well as nitrogen (N), magnesium, and zinc [10, 11]. In wheat and barley, contribution of spikes to grain filling has a range of 10% to 76% [12]. In rice, gross photosynthetic rate of the panicle is 30% of that of the flag leaf, and it was estimated that panicle photosynthesis contributed 20% to 30% of the dry matter in grain [13]. Thus light interception of the ear or panicle should be integrated into the breeding programmes aiming for source-sink balance.

Technical advances in high throughput field phenotyping on a breeding scale in realistic field environments have strengthened the power of physiological breeding [4]. Concurrently, methods for data mining of the big data acquired by various phenotyping platforms are developed. Among them, deep learning has been widely used in image data analysis to explore spatial and temporal information concerning crop growth and development [14]. Leaf area and number indicate the photosynthetic capacity of the crop canopy, and the precise segmentation and counting of leaves has been one of the objectives of image processing. Studies have resulted in robust methodology of deep learning for quantifying leaf number from 2D images [15] and 3D images [16–18], providing effective tools for growth estimation and yield prediction of crop plants. Spike (wheat) or panicle (rice) number per square meter is the key component of cereal grain yield. Numerous attempts have been made to segment and count this reproductive organ accurately in rice [19–21] and wheat [22–24]. Collectively, these robust, low-cost and efficient methods to assess the number of economic organs are of high relevance for phenotyping efforts towards increases in cereal grain yield. However, to our knowledge, method development is still necessary to simultaneously extract both leaf and panicle from the background of a field crop population, as required by the breeder to adopt physiological strategies to balance source and sink.

In this study, we applied a deep learning approach to accurately extract leaf and panicle image data and subsequently calculate the leaf to panicle ratio (LPR) of rice populations during grain filling stage. Of note, the LPR is proposed as a proximate estimation of the distribution of light interception between leaf and panicle, with an assumption that the light captured by the camera is the sunlight directly reflected by the leaf or panicle. Images of training dataset were captured in the field experiments, with large variations in camera shooting angle, the elevation angle and the azimuth angle of the sun, rice genotype, and plant phenological stages. Accurately labeled by manually annotating all the panicle and leaf regions, the resulting dataset were used to train models of FPN-Mask (Feature Pyramid Network Mask) [25], consisting of a backbone network and a task-specific sub-network. The model with the highest accuracy was then selected to study the variations in LPR among 192 japonica rice germplasms and among agronomical practices. Our aim was to provide a high throughput protocol and new physiological trait for breeders to select superior rice cultivars as well as for agronomists to precisely manage field crops that have a good balance of source and sink.

## Methods

**Fig. 1:**
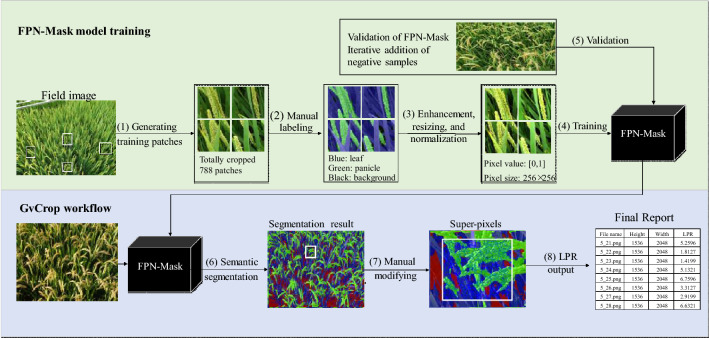
The overall work-flow of panicle-leaf quantification. The upper shows the training procedure of the FPN-Mask model implemented in this study. The bottom depicts the GvCrop working procedure to calculate the LPR (leaf to panicle ratio). (1) Generating 1896 patches by random manual cutting. (2) Manual labelling of every pixel to panicle, leaf and background. (3) Brightness enhancement of patches, normalization to [0, 1] and resizing to 256 × 256 pixels. (4) Training the FPN-Mask model. (5) Daily validation of FPN-Mask with field images and iterative addition of negative samples. (6) Integration of the saved model to semantic segmentation of field images by GvCrop. (7) Manual modification of the predicted result by super-pixel segmentation method integrated in GvCrop

We explored an end-to-end semantic segmentation method to label each pixel as panicle, leaf or background automatically under natural field conditions, and then generated the leaf to panicle ratio (LPR) by division of the number of pixels assigned for each class in each field image. Figure 1 shows the overall work-flow of this method, including two parts. Part 1 is the offline training workflow, with the aim of building a deep learning network called FPN-Mask to segment panicle and leaf from field RGB images. Part 2 is the procedure of GvCrop to develop a software system for calculating LPR.

### Experimental setup

In 2018, plots of ongoing field experiments at Danyang (31°54′31″N, 119°28′21″E), Jiangsu Province, China were selected to take pictures for the training dataset. Of note, these experiments were not specially designed for a phenotyping study. In brief, plant materials of these experiments were highly diverse in genotypic variation, containing seven main japonica cultivars of Jiangsu and 195 mutants with contrasting agronomical traits as reported by Abacar et al. [26]. Further, the seven cultivars had two sowing dates, resulting in obviously different phenotypes for a certain genotype. Thus the diversity in plant architecture and canopy structure of tested materials can provide as many kinds of phenotypes as possible for image analysis.

In 2019, three experiments were conducted to test and apply the proposed FPN-Mask model. (1) Genotypic variations in LPR. A total of 192 mutants were investigated. The plot area was 2.4 m × 1.4 m with a row spacing of 30 cm and plant spacing 20 cm. Nitrogen, phosphate (P_2_O_5_) and potassium (K_2_O) fertilizers were applied at a rate of 240 kg ha^−1^, 120 kg ha ^−1^ and 192 kg ha^−1^, respectively, and were equally separated into basic fertilizers (before transplanting) and topdressing (at 4th leaf age in reverse order). (2) N fertilization effects on LPR. A japonica rice cultivar, Wuyunjing 30, was selected for field experiments with a randomized complete-block design. It had three replications, with a plot area of 2.4 m × 1.4 m. Total N fertilizer was 240 kg ha^−1^ N, and two N fertilization modes with different base/topdressing ratios were applied: (1) N5-5: base/topdressing, 5/5; (2) N10-0: base/topdressing, 10/0. (3) Regulation of plant growth regulators on LPR. Solutions of 100 µM gibberellin, 100 µM uniconazole, 25 µM 2, 4-epibrassinolide, 25 µM brassinazole as well as the control, water, were prepared in distilled water with 0.5% TWEEN-20. One cultivar, Ningjing 8, from the N treatment was used as material. Spraying was conducted at the rate of 500 mL m^−2^ after sunset, with three times starting at booting stage on August 22 and with a 2-day interval.

**Fig. 2:**
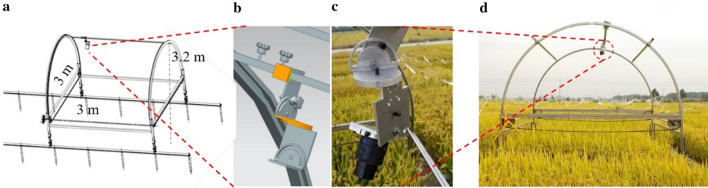
Dynamic canopy light interception simulating device (DCLISD). **a** sketch design of DCLISD. **b** sketch design of unit for simulating the sun’s trajectory by two angles, the elevation and the azimuth angle. **c** RGB camera mounted on the simulation unit; **d** working scene of DCLISD

In addition, a dynamic canopy light interception simulating device (DCLISD) was used to capture images that reflect the sun’s perspective (Fig. 2). The bottom part of it consists of four pillars with wheels and the upper is comprised of two arches consolidated by two steel pipes, and a moveable rail for mounting the RGB camera. The sun’s trajectory is simulated by two angles, the elevation angle and the azimuth angle, calculated according to the latitude, longitude, as well as the growth periods at the experimental site.

### Image acquisition

**Fig. 3:**
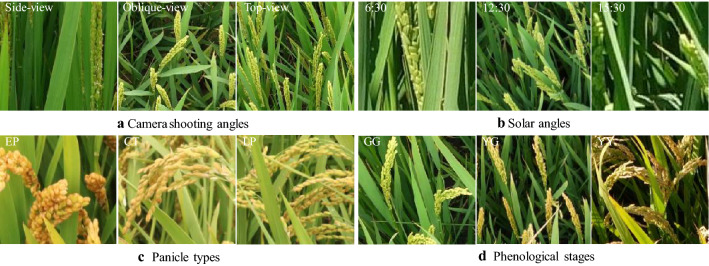
Images selected as training dataset under different field conditions in 2018. **a** camera shooting angles, including side-view (180°), oblique-view (45°) and top-view (90°); **b** solar angles in the morning (5:30 a.m.–10:00 a.m.), at noon (11:00 a.m.–13:00 p.m.) and in the afternoon(15:30 p.m.–18:30 p.m.); **c** cultivars with three panicle types, EP (erect panicle), CT (chicken toe), and LP (loose panicle), according the classification by Zhang et al. [27]; **d** phenological stages, GG (Green panicle with Green leaf) at early, YG (Yellow panicle with Green leaf) at middle, and YY (Yellow panicle with Yellow leaf) at late stage of grain filling

Images of the training dataset were captured in the field experiments in 2018, reflecting the large variations in camera shooting angle, the elevation angle and the azimuth angle of the sun, rice genotype [27], and plant phenological stages (Fig. 3). Images for validation and application of the proposed model were acquired in 2019. For the three treatments of genotypes, N fertilization, and spraying, an angle of 40° was selected for the tripod. The height of the camera (Canon EOS 750D, 24.2 megapixels) was 167.1 cm, the average height of a Chinese adult, and the distance between the central point of the target area and vertical projection of the camera on the ground was 90 cm. The camera settings were as follows: focal length, 18 mm; aperture, automatic; ISO, automatic; and exposure time, automatic. In the experiment with DCLISD, the camera model was SONY DSC-QX100, with settings were as follows: focal length, 10 mm; aperture, automatic; ISO, automatic; and exposure time, automatic.

### Dataset preparation

#### Training dataset

Taking into consideration camera angle, solar angle, panicle type and growth stage (Fig. 3), we prepared a training dataset with 360 representative images from the 2018 dataset (Additional file 1: Table S1). The GG (Green panicle with Green leaf) growth stage, YG (Yellow panicle with Green leaf) growth stage and YY (Yellow panicle with Yellow leaf) growth stage were represented by 113, 104, and 143 images, respectively. Figure 1(1–3) shows the preparation of training data. Considering that the original size of these field images is as large as 4864 × 3648 pixels, they were cropped to a size between [150,150] and [600,600] using the Paint.Net software. After obtaining these patches, we labeled pixels of each patch as panicle, leaf and background manually using the Fluid Mask software. Finally, a total of 1896 representative patches were selected as the final training sample set. Among them, 1210 samples were added continuously during the late daily tests of the model. Further, to increase the diversity of the training dataset and avoid overfitting, we performed basic data enhancements to the training set, including random horizontal/vertical flips, rotations by 90°, and histogram equalizations. To reduce illumination effects, we performed random brightness enhancements on the image. All the input images were resized to 256 × 256 pixels. And for a faster and more stable training model, all the input images were normalized to [0,1] [28, 29].

#### Testing dataset

We divided all 2018 collected images into three groups based on rice growth stage. From each group, we randomly selected 35 testing images and finally selected 105 images as the testing dataset (Additional file 1: Table S2). These selected testing images included extraneous objects, such as tracks, chains, neighbor plots and color-charts.

### Network structure

**Fig. 4:**
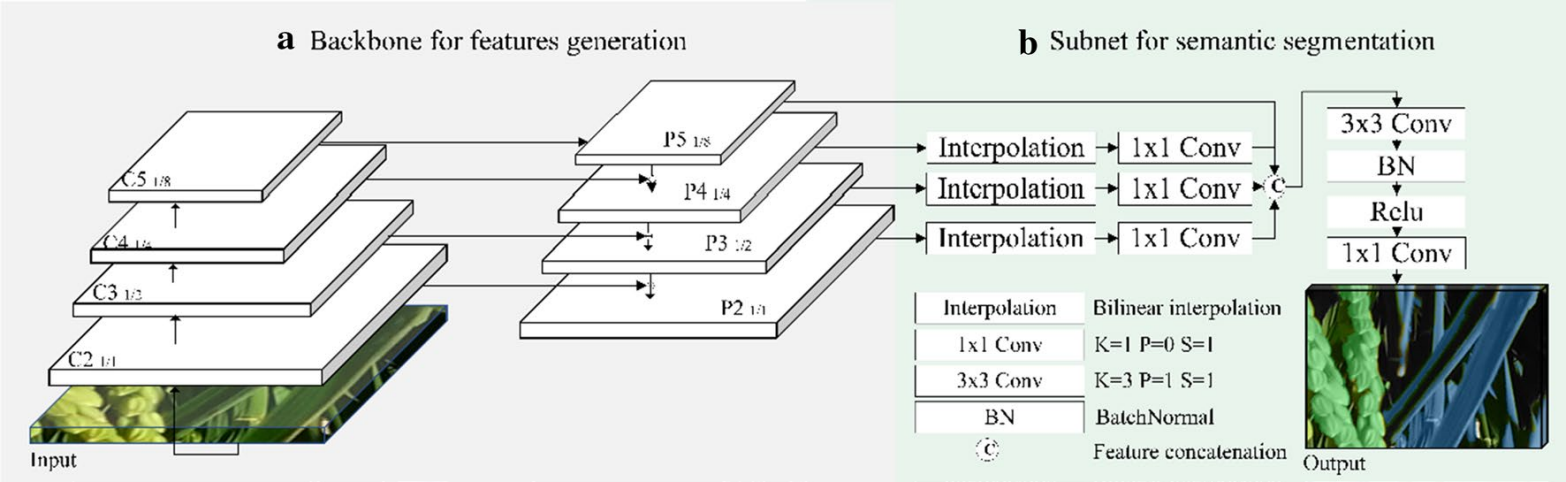
Network architecture. The input layer is a normalized patch, shown in color images. The output consists of the segmented pixels in different colors: green (panicle), blue (leaf), and black (background)

In this study, we proposed a deep learning-based method for rice panicle segmentation, called FPN-Mask. The method consists of a *backbone network* and a *Task-specific subnetwork*. The Feature Pyramid Network (FPN) [25] was selected as the backbone network for extracting features over the entire input data. Originally designed for object detection, it has the advantages of extracting a multi-level feature pyramid from an input image with a single scale. The subnetwork is referenced from the Unified Perceptual Parsing Network [30], which performs semantic segmentation based on the output of the backbone network (Fig. 4).

*Backbone network for feature extraction*: The FPN [25] is a standard feature extractor with a top-down architecture and lateral connections. The top-down architecture is based on Residual Networks (ResNet) [31], with four stages denoted as C_2_, C_3_, C_4_ and C_5_, respectively. We denoted the last feature map of each stage in ResNet as {C_2_, C_3_, C_4_, C_5_}. In our backbone network, we removed the global max pooling layer before C_2_, because it will drop out semantic information. Therefore, the rates of each stage {C_2_, C_3_, C_4_, C_5_} were down-sampled from {4,8,16,32} to {1,2,4,8}. The down-sampling rates of the feature maps derived by FPN {P_2_, P_3_, P_4_, P_5_} were {1, 2, 4, 8}, respectively, meaning that the size of P_2_ is the same as the original image size of 256 × 256, while that of P_5_ is 32 × 32. The number of feature maps output for each stage in ResNet is equal to 32.

*Subnetwork for semantic segmentation*: the subnetwork is based on the multi-level features extracted from the backbone network. Each level of the features will be fused together as an input feature map for semantic segmentation, which has been proved to outperform that only using the highest resolution feature map [30, 32]. To up-sample the low-level feature maps {p_3_, p_4_, p_5_} to get the same size feature as the original image, we directly adopted the bilinear interpolation layer instead of the time-consuming deconvolution layer, and attached a $1\times 1$ convolution layer followed by each interpolation layer to refine the interpolation result. After up-sampling, different levels of the features were concatenated as the final semantic feature. The concatenated multi-level features were convoluted by a $3\times 3$ convolution layer to refine the result and a $1\times 1$ convolution layer to reduce the channel dimensions. The $3\times 3$ convolution layer was attached to a batch normal layer and a relu layer. Finally, we obtained a 3-channel semantic segmentation result, representing background, leaf and panicle, respectively.

### Loss function for semantic segmentation

The cross-entropy loss function is a standard classification method [33]. In practical application, due to the uneven number of pixels from different categories, the loss calculated by the cross-entropy loss function is not realistic [34]. Thus we used the focal loss, which is specifically designed to solve the imbalance problem by focusing on the more difficult classification locations through changing the weight of different categories [34].

### Training

We experimented with ResNet-18 as the FPN backbone. All convolutional layers were initialized as in He et al. [35]. Batch layers were simply initialized with bias $b = 0$ and weight $\sigma = 0$. The mini-batch size was 24, optimization was based on the Adam method, and the training process lasted for 7 days with the base learning rate of 0.001. All the training were conducted on a high-performance computer with Intel 3.50 GHz processor and 128 GB of memory. Two NVIDIA 1080 GeForce graphics processing unit (GPU) has a 12 GB memory used to accelerate the training of our model.

During training, we tested the model performance with all the collected images and selected supplementary training samples for the images that did not perform well to make sure that the training samples covered all the cases of the 6 GB images obtained in 2018 (except the 90 testing images). Sixty images generating 302 patches were added as supplementary training samples, being about 40 samples per day. The performance standards (good or bad) were judged by observation. The training continued until the testing performance of all images visually met the accuracy requirements and the loss function curve was smooth without fluctuations.

### PostProcess

Although a deep network is well suited for processing semantic segmentation problems, errors can not be completely avoided. To lessen the influence of segmentation errors, we further developed a tool, called GvCrop, for manually modifying the auto segmentation results. The software does not only integrate the pixel-wise segmentation method (Fig. 1(6)), but also integrates the ability to modify the segmentation results by human interaction (Fig. 1(7)). Because pixel-level labelling of the wrong location is time consuming, processing the image regions with homogeneous characteristics instead of single pixels can help us accelerate the manual labelling speed (Fig. 1(7)). According to the image color space and boundary cues, we used the gSLICr algorithm to group pixels into perceptually homogeneous regions [36]. gSLICr is the Simple Linear Iterative Clustering (SLIC) implemented on GPU using the NVIDIA CUDA framework, 83 × faster than the SLIC CPU implementation [37]. The gSLICr has three parameters: S, C and N. S stands for super pixel size, C the compact coefficient degree, and N the number of iterations. In this study, S was set to 15, C to 0.2, and N to 50. After super pixel segmentation, users can modify the auto-segmentation results based on super pixels.

### Accuracy assessment

To quantify the performance of the proposed method, we employed three standard metrics to quantify semantic segmentation tasks. (1) Pixel Accuracy (P.A.) indicates the proportion of correctly classified pixels to the total number of pixels. (2) IoU indicates the Intersection-over-union between the ground truth and the predicted pixels, averaged over all classes. (3) Area under the receiver operating characteristic (ROC) curve [38].

1 $$P.A. = \frac{{\sum }_{i}^{n}{p}_{ii}}{{\sum }_{i=0}^{n}{\sum }_{j=0}^{n}{p}_{ij}}(1)$$

2 $$mIoU =\frac{1}{n+1}\times \frac{{\sum }_{i}^{n}{p}_{ii}}{{\sum }_{i=0}^{n}{p}_{ij}+ {\sum }_{j=0}^{n}{p}_{ji}-{p}_{ii}}$$

  where *n* is the number of classes, ${p}_{ij}$ is the number of pixels of class *i* predicted to belong to class *j*, ${p}_{ii}$ is the true positive, ${p}_{ij}$ is the false negative, ${p}_{ji}$ is the false positive, ${p}_{jj}$ is the true negative.

### Calculation of leaf-panicle ratio (LPR)

The software GvCrop was developed to calculate LPR based on the quantity of pixels contained in the leaf (L) and panicle (P) regions in an image and is calculated as: LPR = L/P.

## Results

### Accuracy verification

**Fig. 5:**
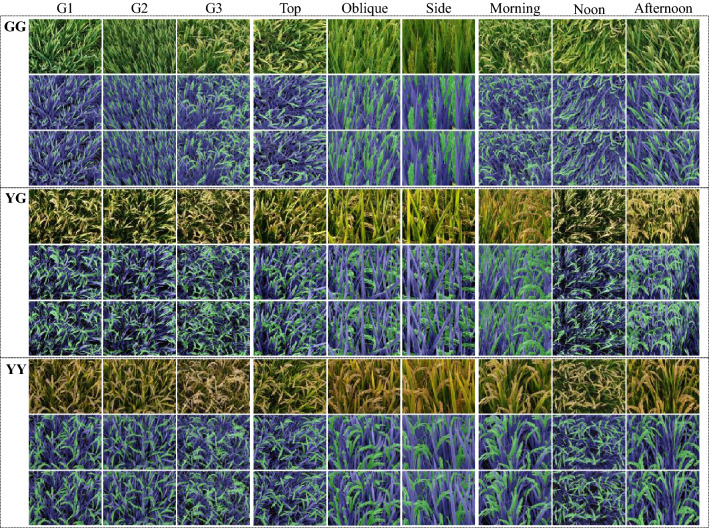
FPN-Mask segmentation results for 27 selected samples at three growth stages of GG, YG, and YY. At each stage, there are three blocks with 9 images from the left to the right, showing differences in genotype (G1, G2, and G3), camera angle (top, oblique, and side views), and solar angle (morning, noon, and afternoon), respectively. Within each block, rows from upper to bottom show the original image, the manually labeled ground truth, and the predicted results, respectively

The semantic segmentation of the 105 field images was assessed both visually (Fig. 5) and quantitatively (Table 1). In addition, we added extra datasets that include non-vegetated objects to evaluate the performance of our proposed method. Fifteen field images at 3 growing stages, 5 images per stage, were added to the testing dataset. The segmentation results using FPN-Mask segmentation method were shown in Additional file 1: Fig. S1, and the corresponding accuracy assessment results were presented in Table 1.

**Table 1:** Accuracy assessments of the automatic leaf-panicle segmentation results

ID	GG		YG		YY	
	mIoU	PA	mIoU	PA	mIoU	PA
1	0.861	0.937	0.846	0.912	0.900	0.950
2	0.851	0.945	0.860	0.921	0.899	0.952
3	0.840	0.946	0.830	0.902	0.866	0.933
4	0.849	0.958	0.844	0.915	0.861	0.934
5	0.842	0.959	0.845	0.919	0.854	0.936
6	0.837	0.954	0.846	0.921	0.850	0.940
7	0.833	0.954	0.850	0.927	0.856	0.944
8	0.822	0.948	0.847	0.923	0.837	0.933
9	0.825	0.950	0.847	0.924	0.842	0.936
10	0.881	0.987	0.887	0.959	0.890	0.945
11	0.869	0.984	0.864	0.943	0.874	0.931
12	0.857	0.978	0.855	0.934	0.887	0.942
13	0.846	0.979	0.845	0.925	0.889	0.941
14	0.837	0.978	0.841	0.928	0.881	0.935
15	0.847	0.976	0.851	0.936	0.880	0.938
16	0.851	0.978	0.856	0.938	0.885	0.943
17	0.852	0.978	0.860	0.942	0.872	0.937
18	0.863	0.974	0.863	0.946	0.875	0.938
19	0.825	0.962	0.867	0.948	0.880	0.942
20	0.824	0.961	0.871	0.949	0.873	0.938
21	0.824	0.961	0.871	0.950	0.871	0.938
22	0.831	0.960	0.870	0.949	0.871	0.939
23	0.838	0.959	0.868	0.947	0.874	0.941
24	0.837	0.960	0.876	0.948	0.874	0.942
25	0.836	0.961	0.880	0.948	0.874	0.942
26	0.833	0.962	0.879	0.947	0.874	0.942
27	0.833	0.963	0.877	0.947	0.872	0.940
28	0.832	0.962	0.877	0.947	0.871	0.940
29	0.831	0.962	0.869	0.942	0.870	0.941
30	0.833	0.963	0.867	0.942	0.871	0.942
31	0.889	0.966	0.931	0.974	0.934	0.986
32	0.879	0.968	0.934	0.973	0.953	0.986
33	0.901	0.970	0.934	0.973	0.960	0.985
34	0.914	0.973	0.941	0.975	0.968	0.986
35	0.912	0.972	0.939	0.974	0.976	0.989
Mean	0.850	0.964	0.871	0.941	0.970	0.987
Min	0.822	0.937	0.830	0.902	0.837	0.931
Max	0.914	0.987	0.941	0.975	0.976	0.989
Std	0.025	0.012	0.014	0.014	0.014	0.005

*GG* green panicle with green leaf, *YG* yellow panicle with green leaf, *YY* yellow panicle with yellow leaf, *mIoU* mean intersection-over-union, *PA* pixel accuracy, *Min* minimum, Max maximum, *Std* standard error of mean

**Fig. 6:**
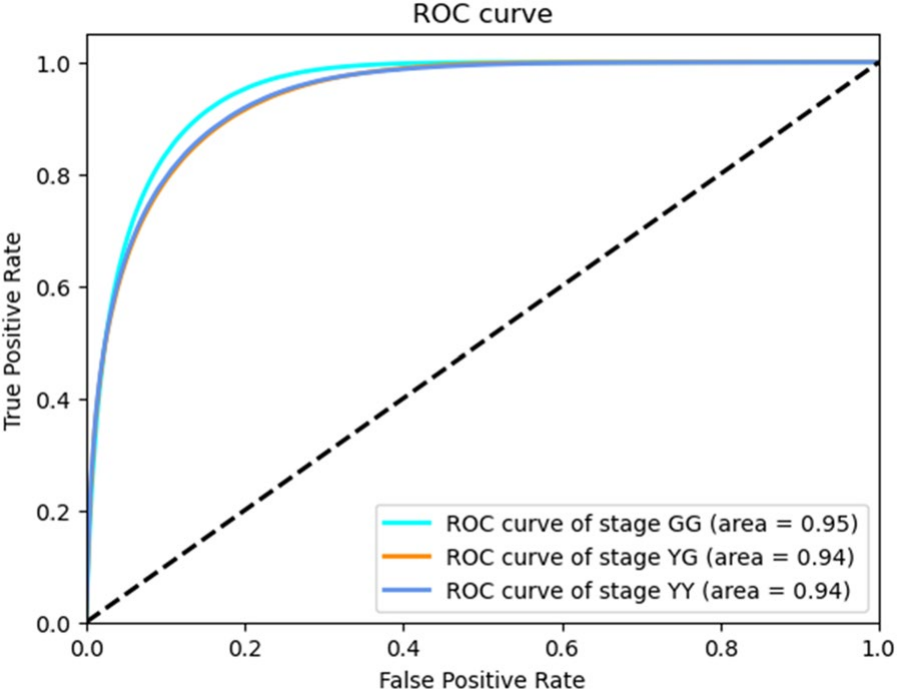
ROC curves and the area under the ROC curves for test dataset at three growth stages of GG, YG, and YY. ROC, receiver operating characteristic

Figure 5 shows some examples of semantic segmentation results. Visual assessment suggested that the tested results and real data were very similar in different conditions. However, we still found some subtle segmentation errors: (1) The background and shadow pixels of the leaves were very similar visually, resulting in some shadow pixels being misclassified as background; (2) The segmentation was a little poor at the edges of the plant parts, with pixels at the junction between leaf and panicle being misclassified into error categories; (3) Some scattered small patches on the leaves were misclassified as panicle. The area under the ROC curve by using our model with the testing datasets were 0.95, 0.94 and 0.94 for GG, YG, and YY, respectively (Fig. 6).

Table 1 provides a quantitative evaluation of the complete test set, showing the high accuracy of all the testing images. Differences between images and growth stages were quite small. Additional file 1: Table S3 shows the model can reach 99% accuracy for panicles pixels, followed by leaf pixels (97.6% to 98.3%), while the worse was for background pixels, ranging from 81.4 to 89.4%.

The efficiency of a training model can also be described in terms of training data loss. Additional file 1: Fig. S2 exhibits that there was a rapid decline in loss over subsequent epochs of training, although the loss was initially high. To avoid overfitting and improve the robustness of our model, we iteratively added samples to the training dataset (Fig. 1(5)) and performed basic data enhancements randomly to the training set before putting it into the model.

### Verification and application of the FPN-Mask model

The most important output of this FPN-Mask model is to estimate the distribution of light interception between leaf and panicle. Using GvCrop, we calculated the LPR values of the crop stand for various field experiments and detected large spatial and temporal variations as well as genotypic differences. Overall, these results suggest the feasibility of the model in detecting and quantifying crop performance under field conditions.

#### Daily changes of LPR

**Fig. 7:**
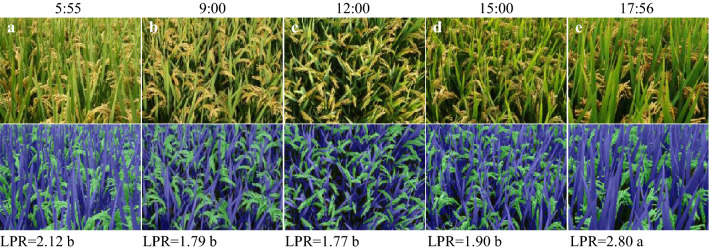
Daily variations in LPR. **a** 5:55 a.m.; **b** 9:00 a.m.; **c** 12:00 a.m.; **d** 15:00 p.m.; **e** 17:56 p.m.. Pictures were taken on October 3, 2019 at Danyang station. Cultivar used was Wuyunjing 29. Mean values with different letters are significantly different according to the shortest significant ranges (SSR) test (P < 0.05)

LPR showed an obvious pattern of daily change, being higher after sunrise and before sunset but lower at noon (Fig. 7). The larger values of LPR in the morning or afternoon can be explained by the shading of leaves when the solar angle of incidence is lower.

#### Genotypic variations in LPR

**Fig. 8:**
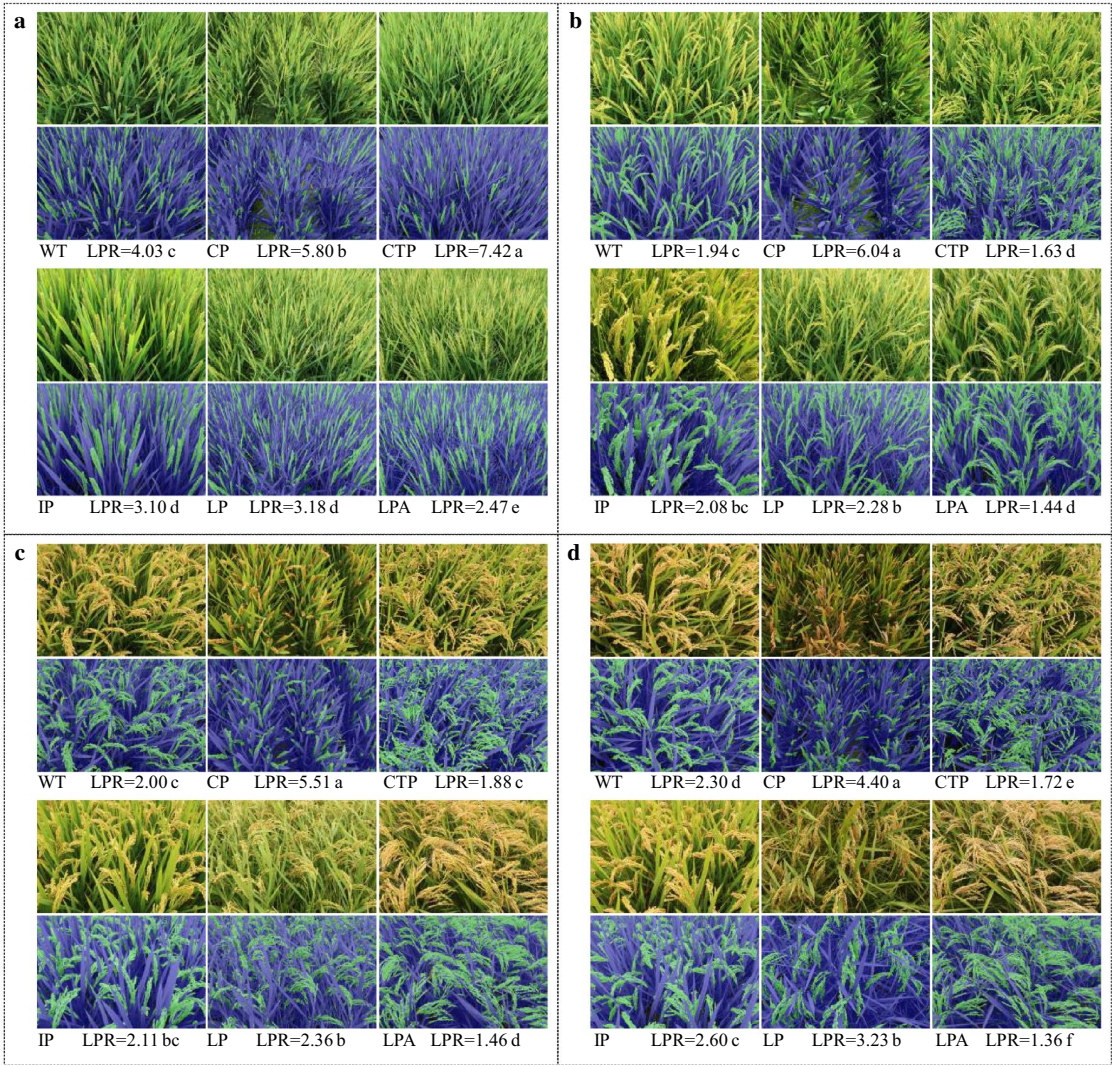
LPRs of cultivars with six panicle types at different growth stages. **a** 0 DAA (days after anthesis); **b** 15 DAA; **c** 30 DAA; **d** 45 DAA. *WT* wild type, *CP* compact panicle, *CTP* chicken toe panicle, *IP* intermediate panicle, *LP* loose panicle, *LPA* loose panicle with awns. The CTP is a special type of LP with more secondary branches on the upper part of the axis, with the lower rachis branches being curved into different directions similar to a chicken foot

Large genotypic differences in LPR were detected among the 192 mutants, ranging from 1.37 to 5.60 (Additional file 1: Table S4). As shown in Fig. 8, the six panicle types showed marked differences in LPR. Generally, cultivars with compact panicle (CP) had the highest value, while those with loose panicle and awns (LPA) had the lowest. The former can be associated with the high density of spikelets on the panicle that caused smaller panicle area. The latter can be explained by the large panicle area due to sparse spikelets. Temporal variations of LPR were revealed showing a diminishing trend from the early stage to the late stage of grain filling. This means the relative area of leaf was reduced, as it is partly due to the increased area of panicle that changes its shape from erect and dense at early stage to loose and drooping at late stage.

#### N effect on LPR

**Fig. 9:**
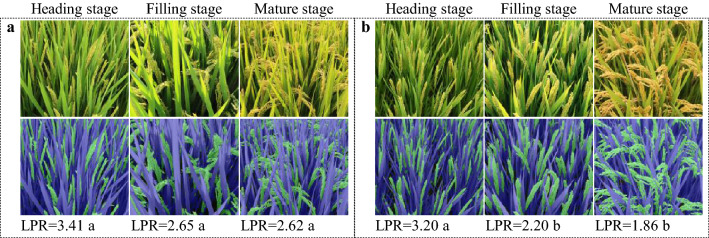
Effect of N fertilization modes on LPR during grain filling. Japonica rice cultivar Wuyunjing 30 is used for representation. A, N5-5, N fertilization treatment with a base/topdressing ratio of 5:5; B, N treatment of N10-0

N fertilization mode exerted substantial influence on LPR. On average, N topdressing of the N5-5 increased LPR by 0.45 and 0.76 at middle and late stage, respectively, compared with N10-0 (Fig. 9). The promoting effect of N topdressing is associated with the elongation of flag leaf (Fig. 9). Similarly, LPR decreased gradually as grain filling progressed for both N treatments.

#### Modification of LPR by plant growth regulators

**Fig. 10:**
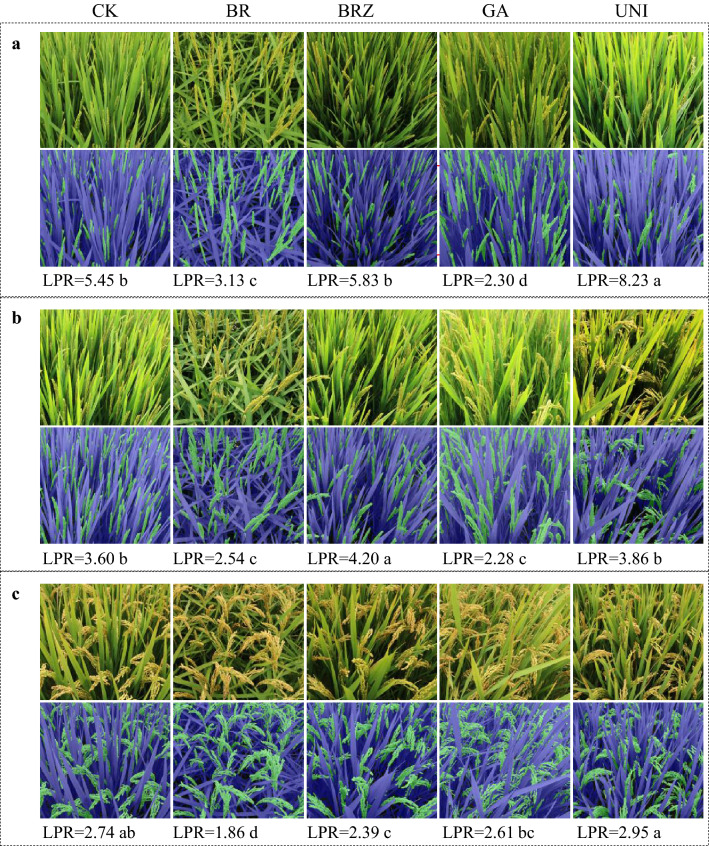
Response of LPR to plant growth regulators. Ningjing 8 is used for representation. **a** Heading stage; **b** grain filling stage; **c** mature stage. CK, water spraying; BR, brassinolide; BRZ, brassinazole; GA, gibberellin; UNI, uniconazole

Plant growth regulators like brassinolide, brassinazole, gibberellin, and uniconazole obviously reshaped the canopy architecture (Fig. 10). The effects of these regulators agreed well with their well-documented phenotypes, for example, the drooping flag leaf caused by brassinolide spraying [39, 40] and the elongated upper internode caused by gibberellin [41]. More importantly, LPR can be either up-regulated or down-regulated by these regulators, depending on growth stages. As shown in Fig. 10, LPR at grain filling stage was increased by brassinazole and uniconazole, whereas reduced by brassinolide and gibberellin. In addition, the degree of increase or decrease depended on regulators, with uniconazole having the most significant influence.

## Discussion

### Weakness of the methodology and improvement

In this study, we built a robust and highly accurate deep learning network, FPN-Mask, which can easily segment panicle, leaf and background at a pixel level from a field RGB image. We also developed the GvCrop software, which not only included some basic image processing functions such as I/O, cut, rotation, zoom in/out, translation, but also integrated the above-mentioned auto semantic segmentation method, manual modification of auto-segmentation result function and export of LPR report function.

This work represents a proof of concept that deep learning can be used for accurate organ level (panicle, leaf) pixel-wise segmentation of field images. The LPR proposed is a novel trait in plant biology, and it is the deep learning method that make it possible to be detected and estimated. Deep learning has been applied to segment plant organs like leaf [15–18] and spike/panicle [19–24], with the aim of counting the number of them and thereby estimating crop yield. This study is totally different from the previous ones, because the newly developed trait of LPR here is based on simultaneous segmentation of leaf and panicle, neither counting the number nor separately detecting them. However, there are several challenges to be tackled in the future.

First, segmentation accuracy was quite high for these 6 GB datasets, but if objects were not included in the training dataset, it would have not performed as well. In other words, the robustness of a deep learning model is partially dependent on the diversity of the training dataset. In the future, we will seek to improve the robustness of FPN-Mask by collecting a wider range of field data. Second, the shadow on leaves and background exhibited very similar visual patterns. It is difficult to distinguish red, green and blue in the visible band. The junctions between different parts of plants are also quite difficult to distinguish. This explained most of the low precision for the semantic segmentation, and these types of errors occurred in every image in the testing dataset. Other studies also encountered the same problem [18].

Third, perspective photography can cause the deformation of objects projected into 2D images, which in turn affects the accuracy of LPR. However, our method has an advantage of reliably calculating the relative value of leaf to panicle ratio using 2D photos, on which the leaf and panicle in the 3D stand is compressed proportionally according to the imaging principle of the camera. Recently, light detection and ranging (LiDAR) has shown its advantages for showing high resolution 3-dimensional (3D) structural information of terrain and vegetation [42–44] and the advantage for segmentation of plant organs [16, 45, 46]. Shi et al. [18] also showed that a multi-view 3D system can avoid these errors. In the future, we will combine plant height provided by LiDAR to texture and color information provided by the RGB image to distinguish object categories more effectively and accurately.

### Significance of LPR for crop breeding and management

To some degree, the essence of crop sciences is the knowledge of selection (by breeders) or regulation (by agronomists) of agronomical traits. Traditionally, crop scientists heavily depend on visual inspection of crops in the field as well as their evaluation of target traits based on their experience and expertise of the crop, which is labor intensive, time consuming, relatively subjective, and prone to errors [14, 47]. In addition, the target traits are mainly morphological traits including leaf senescence, plant height, tillering capacity, panicle or spike size, and growth periods, while fewer physiological traits are monitored and analyzed. With the development of plant phenotyping techniques, image-based methods have been successfully applied to obtain phenotypic data related to crop morphology and physiology [16]. In wheat, high throughput methods for a large array of traits are available for the breeders, including canopy temperature, normalized difference vegetation index (NDVI), and chlorophyll fluorescence [48]. However, the capacity for undertaking precision phenotyping of physiological traits is lagging far behind the requirement of crop sciences.

In this study, we propose a new physiological trait, LPR, based on deep learning. Physiologically, LPR indicates the distribution of light interception within the canopy between the source organ leaf and the sink organ panicle. Historically, breeders and agronomists focused on improvement in source activity, with traits of the leaf such as photosynthesis, erectness, and stay-green as the main targets. On the other hand, the role of panicle was largely overlooked, with less attention except for grain number per panicle or erectness of the panicle [7]. The significance of the panicle has been increasingly recognized in terms of its substantial contribution of carbohydrates, nitrogen, and minerals to grain filling. Therefore, light interception of panicles is dispensable for yield formation, and there should be a suitable LPR value for a crop stand growing in a given ecological condition.

The trait of LPR should provide crop scientists with new insights into the physiological status of the crop stand from the perspective of source and sink balance. For breeders, large genotypic variations in LPR are detected among the 192 germplasms, with a range of 1.37 to 5.60, indicating the possibility to select elite parents for target hybridization and future studies on the morphological and physiological foundations of LPR. For agronomists, LPR is affected by nitrogen fertilization mode, and high yielding practice of N5-5 showed a relatively higher LPR value than that of N10-0, explaining the yield promotion effect of nitrogen topdressing in terms of source-sink relations. Further, LPR was sensitive to foliar application of plant growth regulators like BR and GA, and can be increased by brassinazole and uniconazole, or reduced by brassinolide and gibberellin. Thus it is possible to develop methods for targeted regulation of crop stands with a desirable LPR by chemical intervention. In addition, LPR can be easily measured by digital camera and even a smartphone camera (data not shown). Considering that the measurement of LPR is vulnerable to variations in lighting conditions, we are currently conducting an experiment to identify the timing that can represent the average or general value of LPR for the whole daytime, which could facilitate the use of LPR by crop scientists. Nevertheless, more work is needed when applying LPR in crop breeding or management, in particular elucidating the inherent link between LPR and yield, and proposing a set of suitable LPR values for different environments or plant types.

## Conclusion

The work represents a proof of the concept that the deep learning can achieve high accuracy in simultaneously detecting panicle and leaf data from complex rice field images. The FPN-Mask model is applicable for detecting and quantifying crop performance under field conditions. The proposed trait of LPR displayed large spatial and temporal variations as well as genotypic differences. It was also sensitive to agronomical practices such as nitrogen fertilization and spraying of plant growth regulators. Therefore, LPR, the novel trait indicative of source and sink relation, should provide a high throughput protocol for breeders to select superior rice cultivars as well as for agronomists to precisely manage field crops to have a good balance of source and sink. However, there are several challenges to be handled in future work, in particular combining plant height by LiDAR with the texture and color information from RGB image to distinguish object categories more effectively and accurately.

## Supplementary information

**Additional file 1.** Additional figures and tables.

## Data Availability

The data used in this study is available from the corresponding author on reasonable request.

## Abbreviations

BR: Brassinolide
BRZ: Brassinazole
CP: Compact panicle
CT: Chicken toe
CTP: Chicken toe panicle
3D: 3-Dimensional
2D: 2-Dimensional
DAA: Days after anthesis
DCLISD: Dynamic canopy light interception simulating device
EP: Erect panicle
FPN: Feature pyramid network
FPN-mask: Feature pyramid network mask
GA: Gibberellin
GG: Green panicle with green leaf
GPU: geForce graphics processing unit
IoU: Intersection-over-union
IP: Intermediate panicle
L: Leaf
LiDAR: Light detection and ranging
LP: Loose panicle
LPA: Loose panicle with awns
LPR: Leaf to panicle ratio
Max: Maximum
Min: Minimum
mIoU: Mean intersection-over-union
N: Nitrogen
NDVI: NORMALIZED difference vegetation index
N5-5: N fertilization mode with base/topdressing ratio of 5/5
N10-0: N fertilization mode with base/topdressing ratio of 10/0; Panicle
PA: PIXEL accuracy
ResNet: Residual Networks
RGB: Red–green–blue
ROC: Receiver operating characteristic
ROI: Region of interest
SLIC: Simple linear iterative clustering
Std: Standard error of mean
UNI: Uniconazole
WT: Wild type
YG: Yellow panicle with green leaf
YY: Yellow panicle with yellow leaf
