# Supplementary material for: Leaf to panicle ratio (LPR): a new physiological trait indicative of source and sink relation in japonica rice based on deep learning
Source: Plant Methods. 2020 Aug 26;16:117. doi: 10.1186/s13007-020-00660-y (PMC7449046; doi:10.1186/s13007-020-00660-y)
Supplement: Supplementary file 1 — Additional file 1. Additional figures and tables. [file 13007_2020_660_MOESM1_ESM.docx]

**Fig. S1 FPN-Mask segmentation results for 15 selected samples at three growth stages of GG, YG, and YY.** At each stage, there are three blocks with 5 images from the left to the right, within each block, rows from upper to bottom show the original image, the manually labeled ground truth, and the predicted results, respectively.

**Fig. S2 Training loss curve**. Each epoch has 8192 iterations.

**Table S1 Statistical information of the training dataset**

| Field image | Min | Max | Average Interval |
| --- | --- | --- | --- |
| Growth Age (mm.dd) | 08.30 | 11.02 | 10.00 |
| Azimuth (°) | -111.79 | 107.71 | 54.875 |
| Altitude (°) | 0.62 | 78.39 | 19.44 |
| Time (hh: mm) | 5:30 | 18:30 | 3.75 |
| Variety | 9 |  |  |

**Table S2 Statistical information of the testing dataset**

| Field image | Min | Max |
| --- | --- | --- |
| Growth Age (mm.dd) | 08.30 | 11.02 |
| Time (hh: mm) | 5:30 | 18:30 |
| Variety | 9 |  |

**Table S3 Pixel accuracy for each class**

|  | Panicle | Leaf | Background |
| --- | --- | --- | --- |
| GG | 0.99 | 0.976 | 0.814 |
| YG | 0.99 | 0.983 | 0.849 |
| YY | 0.99 | 0.977 | 0.894 |

**Table S4 LPR of 192 mutants at 30 DAA**

| Material | LPR | Material | LPR | Material | LPR | Material | LPR | Material | LPR |
| --- | --- | --- | --- | --- | --- | --- | --- | --- | --- |
| DW073 | 1.37 | DW110 | 1.80 | DW165 | 1.95 | DW158 | 2.11 | DW103 | 2.50 |
| DW150 | 1.42 | DW192 | 1.81 | DW041 | 1.95 | DW024 | 2.11 | DW087 | 2.51 |
| DW060 | 1.46 | DW109 | 1.82 | DW010 | 1.95 | DW014 | 2.13 | DW061 | 2.52 |
| DW118 | 1.47 | DW023 | 1.82 | DW179 | 1.96 | DW070 | 2.13 | DW141 | 2.52 |
| DW124 | 1.49 | DW170 | 1.82 | DW102 | 1.96 | DW127 | 2.14 | DW080 | 2.54 |
| DW037 | 1.52 | DW090 | 1.83 | DW089 | 1.96 | DW181 | 2.15 | DW106 | 2.59 |
| DW143 | 1.56 | DW063 | 1.83 | DW105 | 1.96 | DW135 | 2.16 | DW187 | 2.62 |
| DW162 | 1.56 | DW015 | 1.83 | DW007 | 1.96 | DW129 | 2.16 | DW176 | 2.71 |
| DW171 | 1.58 | DW046 | 1.83 | DW017 | 1.97 | DW084 | 2.17 | DW125 | 2.73 |
| DW018 | 1.58 | DW077 | 1.83 | DW008 | 1.99 | DW184 | 2.18 | DW167 | 2.76 |
| DW050 | 1.60 | DW022 | 1.84 | DW190 | 2.00 | DW136 | 2.18 | DW032 | 2.79 |
| DW028 | 1.60 | DW005 | 1.84 | DW096 | 2.00 | DW051 | 2.19 | DW146 | 2.80 |
| DW163 | 1.62 | DW066 | 1.84 | DW166 | 2.00 | DW133 | 2.20 | DW012 | 2.81 |
| DW104 | 1.64 | DW116 | 1.85 | DW016 | 2.00 | DW074 | 2.21 | DW128 | 2.84 |
| DW130 | 1.64 | DW044 | 1.85 | DW140 | 2.01 | DW098 | 2.21 | DW182 | 2.87 |
| DW002 | 1.65 | DW058 | 1.85 | DW078 | 2.01 | DW091 | 2.21 | DW001 | 2.90 |
| DW065 | 1.65 | DW039 | 1.86 | DW123 | 2.02 | DW174 | 2.22 | DW142 | 2.90 |
| DW027 | 1.66 | DW153 | 1.86 | DW030 | 2.02 | DW122 | 2.22 | DW145 | 2.91 |
| DW093 | 1.67 | DW172 | 1.87 | DW115 | 2.02 | DW021 | 2.22 | DW025 | 2.95 |
| DW049 | 1.68 | DW092 | 1.87 | DW079 | 2.02 | DW031 | 2.24 | DW155 | 2.96 |
| DW045 | 1.69 | DW053 | 1.87 | DW114 | 2.02 | DW068 | 2.25 | DW186 | 3.01 |
| DW026 | 1.69 | DW151 | 1.87 | DW175 | 2.03 | DW159 | 2.28 | DW131 | 3.03 |
| DW052 | 1.69 | DW043 | 1.87 | DW100 | 2.03 | DW149 | 2.31 | DW139 | 3.10 |
| DW117 | 1.70 | DW035 | 1.87 | DW147 | 2.03 | DW081 | 2.32 | DW152 | 3.12 |
| DW059 | 1.70 | DW003 | 1.88 | DW071 | 2.03 | DW101 | 2.36 | DW134 | 3.20 |
| DW042 | 1.71 | DW054 | 1.88 | DW188 | 2.04 | DW148 | 2.36 | DW086 | 3.44 |
| DW064 | 1.72 | DW177 | 1.88 | DW072 | 2.04 | DW111 | 2.36 | DW085 | 3.47 |
| DW038 | 1.72 | DW154 | 1.88 | DW013 | 2.05 | DW144 | 2.36 | DW156 | 3.83 |
| DW029 | 1.72 | DW057 | 1.88 | DW132 | 2.06 | DW113 | 2.37 | DW055 | 3.92 |
| DW004 | 1.73 | DW075 | 1.89 | DW056 | 2.06 | DW069 | 2.37 | DW169 | 4.58 |
| DW019 | 1.73 | DW047 | 1.89 | DW180 | 2.07 | DW161 | 2.42 | DW099 | 4.68 |
| DW137 | 1.74 | DW095 | 1.89 | DW009 | 2.07 | DW168 | 2.42 | DW112 | 5.60 |
| DW138 | 1.74 | DW083 | 1.89 | DW107 | 2.07 | DW120 | 2.43 |  |  |
| DW034 | 1.76 | DW160 | 1.90 | DW062 | 2.07 | DW126 | 2.43 |  |  |
| DW097 | 1.76 | DW011 | 1.90 | DW033 | 2.08 | DW121 | 2.44 |  |  |
| DW036 | 1.77 | DW094 | 1.90 | DW189 | 2.08 | DW185 | 2.45 |  |  |
| DW082 | 1.78 | DW191 | 1.90 | DW076 | 2.08 | DW178 | 2.46 |  |  |
| DW006 | 1.79 | DW067 | 1.93 | DW183 | 2.09 | DW020 | 2.47 |  |  |
| DW048 | 1.79 | DW040 | 1.93 | DW164 | 2.10 | DW173 | 2.49 |  |  |
| DW088 | 1.80 | DW119 | 1.94 | DW157 | 2.10 | DW108 | 2.49 |  |  |
